# An Energy Efficient and Reliable Multipath Transmission Strategy for Mobile Wireless Sensor Networks

**DOI:** 10.1155/2022/8083804

**Published:** 2022-08-09

**Authors:** Changlong Sun, Zhengzong Wang, Dongwan Lu, Li Cao, Yinggao Yue, Haihua Ding, Zhongyi Hu

**Affiliations:** ^1^School of Economics and Management, Wenzhou University of Technology, Wenzhou 325035, China; ^2^School of Intelligent Manufacturing and Electronic Engineering, Wenzhou University of Technology, Wenzhou 325035, China; ^3^Intelligent Information Systems Institute, Wenzhou University, Wenzhou 325035, China

## Abstract

Multipath data transmission is a key problem that needs to be solved urgently in wireless sensor networks. In this paper, sensor node failure, link failure, energy exhaustion, and external interference affect the stability and reliability of network data transmission. A multipath transmission strategy for wireless sensor networks based on improved shuffled frog leaping algorithm is proposed. A mathematical model of multipath transmission in wireless sensor networks is established. In the shuffled frog leaping algorithm, combined with the transition probability in the particle swarm optimization algorithm, random individuals in the subgroup are introduced to assist the search when updating the frog individual position, which improves the algorithm's ability to jump out of the local optimum and improves the quality of the optimization algorithm solution. The model is applied to multipath transmission in wireless sensor networks. Then, the shuffled frog leaping algorithm is used to update, divide, and reorganize the sensor nodes to select the optimal node to establish the optimal transmission path and improve the stability and reliability of the network. Simulation experiments show that the algorithm in this paper can ensure the reliability of data transmission, reduce the network packet loss rate and network energy consumption, and reduce the average delay of data transmission.

## 1. Introduction

The Internet of Things (IOT) is based on the Internet, using the radio frequency identification, wireless communication technology (such as Bluetooth, wireless sensor network, mobile network, etc.), infrared sensor, global positioning system, laser scanner, and other technologies to extend the functions of the Internet and connect many items directly to form a network with wider coverage and higher efficiency [1]. Its essence is to use these new technologies to realize intelligent identification, positioning, tracking, monitoring, and management of global items, to achieve real-time information sharing. The technical architecture of the Internet of Things includes the perception interaction layer, the network transmission layer, and the application service layer [2]. Achieving intelligent identification mobile wireless sensor networks (MWSNs) is an autonomous and distributed large-scale of the sensor network. The network topology is dynamically changed, and the tasks are performed in a cooperative manner [3]. It is precisely because of these characteristics that the complexity of the network is greatly increased. For example, the redundant transmission mode adopted in the network leads to a decrease in the reliability and stability of the transmission path of the network [4]. At the same time, the number of redundant transmissions is increased, and due to the uneven consumption of node energy consumption, the energy of some nodes will be completely lost, making the sensor nodes in some areas of the entire network unable to work [5]. Ultimately, it leads to the failure of the sensor network system and affects the network's lifetime. Problems that need to be solved urgently reflect the need for further optimization and development of wireless sensor network technology [6].

### 1.1. Problem Statement and Motivation

Generally speaking, the fault-tolerance capability of a sensor network means that when some nodes or links fail, the network can recover the data transmission or the network structure self-healing to minimize the impact of node or link failure on the function of the wireless sensor network [7]. When a wireless link is disconnected, the routing strategy can automatically find a new path to avoid the disconnected network link. This greatly improves the reliability of the network and is also a key feature of wireless sensor networks. Wireless sensor networks must have strong fault tolerance to ensure high reliability and robustness of the network system. Efficient fault-tolerant routing mechanism is an important part of prolonging the reliability of the network and the system's lifetime.

The multiroute transmission fault tolerance technology establishes a geometric path between the source sensor node and the destination sensor node. By establishing multiple transmission paths, the network bandwidth is limited, and the load balancing ability is improved. The five-layer network protocol transmission of application layer, network layer, data link layer, physical layer, and transport layer is realized through multipath transmission fault tolerance technology and network coding data redundancy transmission technology, combined with energy manager, task manager, and topology manager to coordinate processing control to achieve network transmission reliability and stability. Another common method of multipath transmission is network coding. Network coding is to recompile a data source code to generate data blocks. An information exchange technology that reorganizes the source data after it is transmitted from multiple paths to the destination sensor node. This method combines multirouting and coding technology to improve the reliability of data transmission.

In practical applications, node failure, link failure, and energy consumption have a huge impact on the stability and reliability of multipath data transmission at the network layer. Aiming at this problem that needs to be solved urgently, in this paper, we used the current popular swarm intelligence bionic optimization algorithm and proposed a multipath transmission strategy for mobile wireless sensor networks based on the shuffled frog leaping algorithm optimized by particle swarm optimization. This method combines the classic multipath transmission AODV algorithm, optimizes the data transmission strategy of sensor nodes, locally updates the network, reduces the amount of data transmission, improves the convergence speed of the algorithm, adjusts the threshold selection range, and reduces the space difference of each path to optimize the multipath transmission mode. Ad hoc on-demand distance vector routing (AODV) is a routing protocol used for routing in wireless ad hoc networks (also known as wireless ad hoc networks). It can realize unicast and multicast routing. This protocol is a typical on-demand routing protocol in ad hoc networks. It is an on-demand routing protocol. On demand means that the node will not store the routing information of all nodes in the network. The routing table will be checked only when it needs to transmit data to the nodes. If there is no route, a routing request will be sent to the network. This is the route discovery process to obtain the route to the node [8]. As a general term for algorithms inspired by the biological world, biological intelligence algorithms have the characteristics of robustness, adaptability, and self-healing and are suitable for application in the field of multipath data transmission in wireless sensor networks. This paper combines the characteristics of different types of wireless sensor networks, such as two-level heterogeneous networks and mobile networks, and comprehensively uses multipath routing technology and a variety of biological intelligence algorithms to discuss the routing fault tolerance strategies of these types of networks in the data transmission process [9]. The research results of this thesis can inspire people to develop new fault-tolerant routing methods from the perspective of biological intelligence algorithms for wireless sensor networks to actively promote the development of wireless sensor networks and biological coevolutionary intelligent algorithm theory and technology. The multipath routing fault tolerance strategy proposed in this paper provides a stable and reliable data transmission environment for wireless sensor networks, an efficient multipath routing recovery mechanism, improves the robustness and reliability of the network, and prolongs the network lifetime.

### 1.2. Contribution

In this work, a multipath transmission strategy for mobile wireless sensor networks based on the shuffled frog leaping algorithm optimized by particle swarm optimization (PSO-SFLA) is proposed. In comparison with the current general selection approaches, the main contributions of our work in this paper can be summarized as follows.

1. Characterize the issues of the multipath transmission strategy for mWSNs, and classify the current multipath transmission strategy.
2. Propose a multipath transmission strategy for mobile wireless sensor networks based on the shuffled frog leaping algorithm optimized by particle swarm optimization (PSO-SFLA).
3. Evaluate the performance of the proposed algorithms by comparing them with the multipath transmission strategy of the PSO, SFLA, and PSO-SFLA algorithm.

## 2. Related Work

The multipath routing transmission mechanism and data encoding and decoding transmission mechanism of mobile wireless sensor network are mainly to improve the stability and reliability of network transmission and improve the fault tolerance of network transmission [10]. This section will introduce the research progress of related research work. Due to the mobility of sink node, the dynamic change of topology, the complex and bad deployment environment of node, and many uncertain factors, the sensor node fails, the quality of data transmission link becomes worse, and the data packet transmission fails, which seriously affects the stability and reliability of network operation and brings great challenges to the adaptability and robustness of the network [11]. The commonly used method is to adopt the fault tolerance strategy, which can improve the operation stability and network reliability of the mobile wireless sensor network. The fault tolerance strategy of MWSNs refers to the interruption of the original data transmission path when a node or communication link in the sensor network fails. The following have become urgent problems that should be solved in current mobile wireless sensor networks: how to quickly restore an efficient and reliable routing transmission path, handle network faults adaptively, quickly, and effectively, and ensure high reliability and accurate operation of the network; how to design an efficient fault-tolerant strategy, provide a stable and reliable data transmission path, ensure the robustness and anti-interference ability of data transmission, and improve network reliability and availability. The fault tolerance of MWSNs mainly includes the design of fault-tolerant protocols at all layers of the network and the multilayer joint optimization control. There are two methods commonly used in network layer routing fault tolerance: multipath routing transmission fault tolerance technology and network coding for redundant transmission of transmitted data. Fault tolerance design mainly focuses on node hardware fault tolerance, coverage fault tolerance, topology control fault tolerance, and routing fault tolerance. Among them, routing fault tolerance is the foundation and focus of MWSN fault tolerance research. At present, the routing fault tolerance methods proposed by domestic and foreign researchers mainly focus on four aspects: link retransmission method, network coding, error correction code mechanism, and multipath data transmission method.

### 2.1. Link Retransmission Method

The wireless communication link failure rate in MWSNs is much higher than that in finite networks and increases with the increase of node transmission hops. The link retransmission mechanism only needs one retransmission to transmit the information to the required node. From the point of view of the validity of MWSN channel usage, link retransmission is an effective method. However, in a special application field, the link retransmission mechanism also has obvious shortcomings. Frequent data retransmission will affect the utilization of the channel, and the sensor node will retain the information to the route reconfirmation of the next hop, which greatly occupies the limited memory of the node, which is obviously not applicable in the application of MWSNs.

In [12], the authors discuss the use of network coding algorithms combined with appropriate retransmission techniques to improve communication reliability in wireless sensor networks (WSN). Reference [13] proposes a flow-centric strategy to reallocate retransmissions flexibly and dynamically between links of multihop flows at runtime. This contribution is complemented by a method for determining the number of retransmissions required to achieve user-specified reliability levels under two failure models that capture common wireless characteristics of industrial environments. Reference [14] developed a retransmission strategy derived from the Pareto principle and the scale-free properties of complex networks. In our revised definition of hop-by-hop reliability, sensors that forward data directly to the central node must perform retransmissions. Reference [15] addresses the degree of irregularity parameters to facilitate adaptation to geographic switching for two types of transmission in distributed systems: hop-by-hop and end-to-end retransmission schemes. The simulations determined results for average packet delay transmission, transmission energy consumption, and throughput. Simulations provide insights into the impact of radio irregularities on neighbor discovery routing techniques for both schemes. Reference [16] analyzes the energy efficiency of unlicensed wireless networks, where retransmissions are possible if a transmitted message is decoded while it is interrupted. After finding the optimal throughput in the presence of retransmissions, we focused on the overall power consumption and energy efficiency of the network and how retransmissions, network density, and outage thresholds affect the network's energy efficiency.

### 2.2. Network Coding

Redundant transmission of network-coded data is to encode the source data packets, transmit the encoded data slices through multiple paths, and decode and reassemble a certain number of encoded data slices into source data packets at the destination node to achieve transmission fault tolerance.

To increase the reliability of data transmission or provide load balancing, [17] proposes a network coding multipath routing algorithm in WSN (NC-WSN). Reference [18] proposes an energy-efficient adaptive data aggregation strategy using network coding (ADANC), which improves the energy efficiency of cluster-based duty-cycle WSNs. Network encoder nodes also act as aggregation points opportunistically according to the level of data correlation. Reference [19] studies network coding as a power minimization technique. Reference [20] proposed a reliable data dissemination protocol AdapCode, which adaptively changes the coding scheme according to the link quality. It uses adaptive network coding to reduce broadcast traffic during code updates. The data packets on each node are encoded by linear combination and decoded by Gaussian elimination. Reference [21] proposed a network coding-based probabilistic routing scheme (NCPR) for wireless sensor networks to alleviate the broadcast storm problem and improve the network coding gain.

### 2.3. Error Correction Code Mechanism

Using the method of reconstructing the original data, the error correction code mechanism can obtain higher network reliability without link retransmission. However, it needs to divide the data packet into various code words. There is a defect in this method. In the selection of the number of information and code words, the number of information cannot exceed the number of bits used to represent the information. At the same time, the number of code words must be less than the capacity used to calculate the external storage space.

Reference [22] proposes a step-by-step method to find suitable error correction codes for WSNs. Several simulations considering different error correction codes show that RS meets the BER and power consumption criteria. Reference [23] studies two ECC algorithms, which are block codes and convolutional codes, for energy, power, and performance. Reference [24] proposed an adaptive error control implementation framework. This approach leverages the capabilities provided by software-defined networking (SDN) and forward error correction (FEC). The framework supports the adaptability of transmitters and receivers. Reference [25] proposes a fast and energy-efficient single error correction-double error correction (SEC-DED) and single error correction-double error correction-double adjacent error correction (SEC-DED-DAEC) codes. The proposed SEC-DED and SEC-DED-DAEC codecs require lower area, latency, and power compared to existing coding schemes. Reference [26] proposed a new adaptive error control (AEC) algorithm. Adaptive error control adaptively changes the error correcting code (ECC) based on the channel behavior observed through the packet error rate (PER) in the most recent previous transmission.

### 2.4. Multipath Data Transmission

The multiroute transmission fault-tolerant technology can be divided into methods such as reestablishing multiple transmission paths when a node fails or selecting activation among the established multiple paths. The most important purpose is to extend the network life and improve the reliability and success rate of data transmission. The multipath routing fault tolerance method is to establish multiple data transmission paths between the source node and the destination node. When the sink node moves a certain distance away from the communication range of the node, the interference of external factors or the failure of the node itself causes communication interruption, and then, the current transmission path will become invalid. The method can quickly switch to another transmission path to obtain better fault tolerance and improve the reliability of data transmission. In the multipath transmission method, how to establish multiple reliable transmission paths quickly and efficiently between the source node and the sink is the key to this problem.

In [27], this paper proposes a new multipath reliable transmission method (named RCB-MRT) suitable for edge wireless sensor networks. It adopts redundancy mechanism to realize the reliability of data transmission and adopts concurrent weaving multipath technology to improve the transmission efficiency of data packets. Reference [28] developed a new algorithm called exponential ant colony optimization (EACO) to solve the routing discovery problem in wireless sensor networks after finding the cluster head (CH) using the fractional artificial bee colony (FABC) algorithm. Reference [29] proposes a directed diffusion-based multipath algorithm that enhances multiple routes with high link quality and low latency. Reference [30] proposed a super cluster head (SCH) selection algorithm between CHs based on fuzzy concepts. A further cost function (CF) is proposed for the mean residual energy and mean end-to-end (ENE) delay and mean transmission reliability (AR) of the multipath routing network.

Based on summarizing the predecessors, this paper adopts a distributed processing method to accurately monitor and measure big data according to the regional structure of the network at each level. The shuffled frog leaping is applied to the transmission mode between the source node and the destination node in the multiroute data transmission mechanism, and the pheromone normalization of the shuffled frog leaping is optimized by particle swarm optimization (PSO-SFLA) to reflect the network path link information volume. According to the improved leapfrog model, the comparison of network connectivity, transmission delay, energy conversion efficiency, and other data was analyzed and simulated, and the feasibility of the improved shuffled frog leaping in its wireless network transmission was verified.

## 3. Mathematical Model

**Figure 1:**
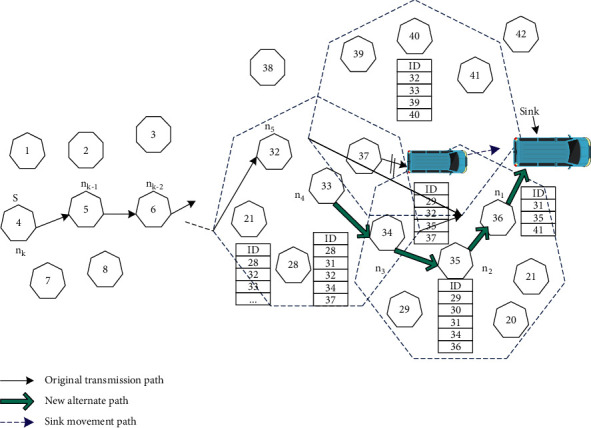
Multipath data transmission process of mobile sink.

In the data transmission of mobile wireless sensor network, the sensor node *n* on the transmission path from the source node to the destination node has a neighbor table, which is used to store the relevant information of neighbor nodes, such as the unique ID number of each node in the network, the node residual energy, communication energy consumption, and transmission delay. When the sensor node *n* receives the data packet forwarded by the neighbor node *n*_*b*_ for the first time, it first registers the information of *n*_*b*_ in the routing table in the transmission path. *N*_1_(*n*_*k*_) is the set of all nodes in the neighbor table of the sensor node *n*_*k*_, and *N*_*h*_(*n*_*k*_) is the set of *N*_*h*−1_(*n*_*k*−1_). The neighbor table of the mobile sink stores the neighbor nodes one hop away from itself. *N*(*n*_sin*k*_) represents the neighbor table stored in the sink node, and the information of the routing table is mainly used to restore the new transmission path. The routing fault-tolerant recovery process of the mobile sink is shown in Figure 1.

The main influencing factors for finding the best alternative transmission path *Q*_*j*_ are the remaining energy of neighbor nodes, the length of the transmission path between adjacent nodes, the network energy consumption, and the transmission delay. Replace the remaining energy Rene(*n*_*j*_^*k*^) of the neighbor node on the path, replace the path length Dist(*e*_*j*_^*k*^) of the effective link between two adjacent nodes on the path, replace the energy consumption Ene(*n*_*j*_^*k*^) and Ene(*e*_*j*_^*k*^) between a single node on the path and two adjacent nodes, and replace a single node on the path and the transmission delays Delay(*n*_*j*_^*k*^) and Delay(*e*_*j*_^*k*^) between adjacent nodes. The fitness fitness(*Q*_*j*_) formula of the best alternative transmission path *Q*_*j*_ is as follows:

(1) $$\begin{array}{c} \mathrm{fitness}\left(Q_{j}\right)=\frac{\sum_{n_{j}^{k}\in Q_{j}}\text{Rene}\left(n_{j}^{k}\right)}{w_{1}f_{1}+w_{2}f_{2}+w_{3}f_{3}},\\ f_{1}=\frac{\sum_{n_{j}^{k}\in Q_{j}}\mathrm{Ene}\left(n_{j}^{k}\right)+\sum_{e_{j}^{k}\in Q_{j}}\mathrm{Ene}\left(e_{j}^{k}\right)}{\sum_{n\in V}\mathrm{Ene}\left(n\right)+\sum_{e\in E}\mathrm{Ene}\left(e\right)},\\ f_{2}=\frac{\sum_{n_{j}^{k}\in Q_{j}}\mathrm{Delay}\left(n_{j}^{k}\right)+\sum_{e_{j}^{k}\in Q_{j}}\mathrm{Delay}\left(e_{j}^{k}\right)}{\sum_{n\in V}\mathrm{Delay}\left(n\right)+\sum_{e\in E}\mathrm{Delay}\left(e\right)},\\ f_{3}=\frac{\sum_{e_{j}^{k}\in Q_{j}}\mathrm{Delay}\left(e_{j}^{k}\right)}{\sum_{e\in E}\text{Dist}\left(e\right)}. \end{array}$$

Among them, the parameter *f*_1_ is the proportion of the total energy consumed by the links contained in a path to the total energy consumed by all the links in the cluster. The parameter *f*_2_ is the total delay of the nodes contained in the path in the total energy consumption of all nodes in the cluster. The proportion of the delay of the parameter *f*_3_ is the proportion of the total length of the links contained in this path to the total length of all links in the cluster. The parameters *ω*_1_, *ω*_2_, and *ω*_3_ are the weights corresponding to the functions *f*_1_, *f*_2_, and *f*_3_, respectively. *ω*_1_+*ω*_2_+*ω*_3_=1, *ω*_1_=0.4, *ω*_2_=0.2, and *ω*_3_=0.4. It can be seen that the fitness represents a better network path.

At the same time, we define the energy model formula of a single sensor node as follows:

(2) $$\begin{array}{c} \text{ene}\left(m,d\right)={\text{ene}}_{tx}\left(m,d\right)+{\text{ene}}_{rx}\left(m\right)\\ =\left(a_{11}+a_{2}d^{n}\right)m+a_{12}m. \end{array}$$

Among them, the parameter *d* is the distance between the node and the next-hop neighbor node, ene_*tx*_(*m*, *d*) and ene_*rx*_(*m*) are the energy consumption of sending and receiving *m*-bit data respectively, and the parameters *a*_11_, *a*_2_, and *a*_12_ are the energy consumption of the transmitting circuit, transmitting amplifier, and receiving circuit, respectively. The parameter *n* is the channel attenuation index. The parameter ene_*DF*_ is defined as the energy consumption for data fusion, and ene_*RT*_ is the energy consumption for updating the routing table. We define the energy consumption of particle update, immune cloning, high frequency mutation, particle selection, and suppression for each iteration of MWSNs in the simulation as ene_*PU*_, ene_*IC*_, ene_*IM*_, ene_*PS*_, and ene_*PR*_, respectively. Therefore, the total energy consumption of MWSNs can be estimated according to the energy consumption of each iteration in each round of simulation.

## 4. Shuffled Frog Leaping Algorithm Optimized by PSO

### 4.1. Shuffled Frog Leaping Algorithm

Shuffled frog leaping algorithm (SFLA) is a swarm intelligence optimization algorithm based on global search proposed by Maaroof et al. in the new century. It can simply and quickly find the optimal solution in the set of feasible solutions of combinatorial optimization [31]. The SFLA algorithm, which is inspired by the frog's search for food, searches for the target based on the cooperative cooperation between the populations [32]. First, different individuals search for information, and then, exchange information in the local scope to construct an information search strategy in the subgroup scope. With the exchange of information within the subgroups, internal evolution is formed, and then, different subgroups are derived, and the information between the subgroups is reanalyzed and exchanged to form a global information exchange [33]. These two approaches interact with the population, acting alternately to solve combinatorial optimization problems. The SFLA algorithm combines the characteristics of the flock foraging method for iteratively finding the optimal solution of the population behavior and the meme algorithm of genetic bionics and is widely used in the traveling salesman problem, function processing, power supply system optimization, machine vision, and so on [34].

The mathematical model and specific calculation process of the hybrid frog leaping algorithm, in a *d*-dimensional target search space, randomly generate *D* frog individuals, that is, the number of solutions of the target optimization function, forming the initial frog population *S*={*X*_1_, *X*_2_,…, *X*_*D*_}. The current position of the *i*-th frog individual is the solution of the current combinatorial optimization problem. Assuming that the current frog individual is *X*_*i*_={*x*_*i*1_, *x*_*i*2_,…, *x*_*i*  *d*_}, the individual fitness function value *f*(*X*_*i*_) of each frog can be obtained by calculating and solving, and then the obtained individual fitness values of the frogs are arranged in descending order. At the same time, referring to the frog group division criterion, the entire frog group is divided into *N* population groups *Y*^1^, *Y*^2^,…, *Y*^*N*^, the frog population is divided into *N* population groups, and parameter *N* is the number of frog population groups. Each subgroup contains *M* frog individuals, which satisfies *D* = *N* × *M*. The frog division criterion is to divide the 1st frog into the 1st subgroup, the 2nd into the 2nd self-group, and so on, until the entire frog group is divided. The following is obtained [35]:

(3) $$\begin{array}{c} Y_{j}=\left\{X_{j+N\left(l-1\right)}\in S\right\},\;1\leq l\leq M,1\leq j\leq N. \end{array}$$

A local optimal search is performed in each frog subgroup, and in each iteration, the following three parameters can be obtained. The optimal individual position *X*_*b*_, the worst individual position *X*_*w*_, and the global best individual position *X*_*g*_ are taken from the group, and then, the worst individual position of the frog is updated. The update strategy is shown in the frog step update formula:

(4) $$\begin{array}{c} Q_{i}=\text{rand}\times \left(X_{b}-X_{w}\right),\;-Q_{\max}\leq Q_{i}\leq Q_{\max}. \end{array}$$

The positions of the frog subgroup individuals are updated according to the following:

(5) $$\begin{array}{c} X_{W}^{\prime }=H_{w}+Q_{i}, \end{array}$$

where rand() is a random number between 0 and 1, and the parameter *Q*_max_ is the maximum step size allowed to update in the frog subgroup. After executing the update strategy of (4) and (5), the new position *X*_*W*_′ is obtained. If the newly obtained position *X*_*W*_′ is better than the previous *X*_*W*_, the frog will replace the previous position *X*_*W*_′ with the previous *X*_*W*_. Otherwise, according to (6), update the strategy transformation and update the step size formula:

(6) $$\begin{array}{c} Q_{i}=\text{rand}\times \left(X_{g}-X_{w}\right),\;-Q_{\max}\leq Q_{i}\leq Q_{\max}, \end{array}$$

(7) $$\begin{array}{c} X_{W}^{\prime }=H_{w}+Q_{i}. \end{array}$$

After the new update, (6) and (7) are executed, the new frog position is obtained, and the same is compared with the previous position to replace the current position, if the frog position has not been changed. Then, a new individual position (the solution of the function) is randomly generated to replace the individual position of the principle frog, and we can obtain

(8) $$\begin{array}{c} X_{w}^{\prime \prime }=\text{rand}\times \left(O_{\max}-O_{\min}\right)+O_{\min}. \end{array}$$

**Figure 2:**
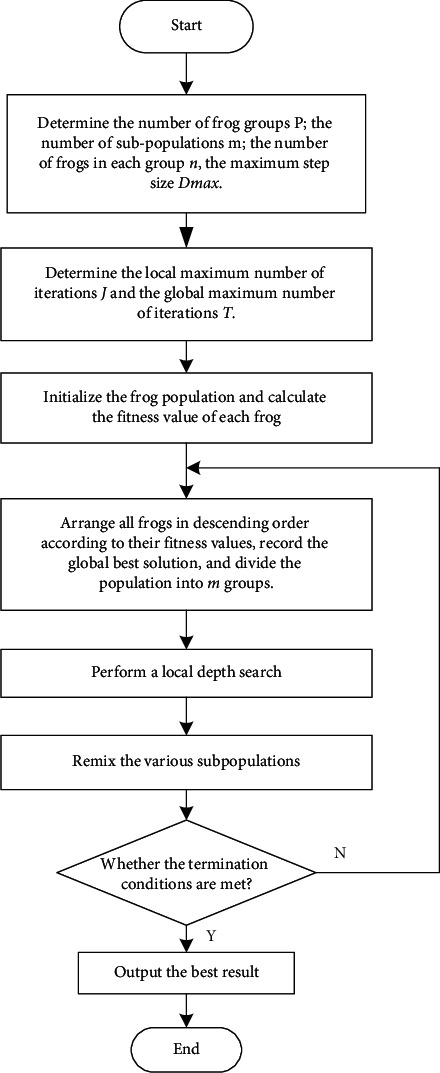
The flowchart of the shuffled frog leaping algorithm.

Among them, the parameter *X*_*w*_^″^ is the current latest position, and the parameters *O*_max_ and *O*_min_ are the maximum and minimum values of the search range in the frog subgroup, respectively. The previously mentioned iterative update steps are repeated continuously until the maximum number of iterations set in the preset subgroup is satisfied. When all subgroups perform local optimal search and obtain the optimal position of individual frogs, global information exchange is carried out. The exchange process involves remixing, sorting, and resubgrouping all frog individuals. Afterwards, a deep local search for the optimal solution (the best position) is performed in each new subgroup, and so on and so forth, until the preset iterative conditions are met. The hybrid leapfrog algorithm is shown in Figure 2.

### 4.2. Shuffled Frog Leaping Algorithm Optimized by PSO (PSO-SFLA)

Combining the characteristics of particle swarm optimization and the shuffled frog leaping algorithm, a new improved SFLA algorithm based on particle swarm optimization is proposed in this paper. Kennedy and Eberhart proposed a new global search method, the particle swarm optimization algorithm. A lot of practice has proved that this method is effective in solving optimization problems. Particle swarm optimization algorithm is a heuristic algorithm with individual improvement, population cooperation, and competition mechanism, which is inspired by the predation behavior of flocks of birds or fish in nature. Each particle updates its position in the decision space at an adaptable rate to bring the particle closer to the required space. The optimal position of each particle and the position information of the optimal particle in the particle swarm jointly guide the search position of the particle swarm. Each particle has a corresponding velocity and position and a fitness value determined by the objective function. Each iteration of the particle in the solution space is based on the currently found better solution to find the next solution. The *i*-th particle is denoted as *X*_*i*_=(*x*_*i*1_, *x*_*i*1_,…, *x*_*i*  *d*_), and it has experienced the best position (with the best fitness value) and denoted as *P*_*i*_=(*p*_*i*1_, *p*_*i*1_,…, *p*_*i*  *d*_), also known as *p*_best_. The best position of all particles in the group in the iteration is represented by the symbol *P*_*g*_, also called *g*_best_, and the velocity of particle *i* is represented by *V*_*i*_=(*v*_*i*1_, *v*_*i*1_,…, *v*_*id*_). The update of the velocity and position of the *i*-th particle in the *d*-th dimension decision space adopts the following expressions:

(9) $$\begin{array}{c} v_{id}\left(t+1\right)=wv_{id}\left(t\right)+c_{1}r_{1}\left(p_{i\;d}\left(t\right)-x_{id}\left(t\right)\right)+c_{2}r_{2}\left(p_{gd}\left(t\right)-x_{i\;d}\left(t\right)\right),\\ x_{id}\left(t+1\right)=x_{id}\left(t\right)+v_{id}\left(t+1\right), \end{array}$$

where *i*=1,2,…, *N*, *d*=1,2,…, *n*. The parameter *w* is the inertia weight, the parameters *r*_1_ and *r*_2_ are two random functions that vary in the range of [0, 1], and the parameters *c*_1_ and *c*_2_ are the acceleration constants, which determine the learning ability of the particle to its own optimal position and the optimal position of the population, respectively.

The steps of calculating the global optimal solution of shuffled frog leaping algorithm optimized by PSO are as follows.

  Step 1: Randomly initialize the position and velocity of each particle of the population on the search space *D*.
  Step 2: For each particle, calculate the fitness function.
  Step 3: Obtain *p*_best_ and *g*_best_ values for the entire population. Compare particle fitness evolution with particle *p*_best_. If the current value is better than *p*_best_, set the *p*_best_ value equal to the current value and the *p*_best_ position equal to the current position in the dimension space.
  Step 4: Compare the fitness value with the overall *p*_best_ value. If the current *p*_best_ value is better than *g*_best_, reset to the current particle value.
  Step 5: Change the velocity and position of the particle according to (4) and (5), respectively.
  Step 6: When the number of iterations of the algorithm reaches the maximum number or meets the minimum error requirement, stop the iteration; otherwise, jump to Step 2.
  Step 7: Search for possible solutions to the population of the frogs, which are sorted in descending order according to their fitness and divided into subsets called memeplexes (*m*).
  Step 8: The *i*-th individual of the frog is denoted as *X*_*i*_=(*X*_*i*1_, *X*_*i*2_,…*X*_*is*_), where the parameter *S* represents the number of variables.
  Step 9: For each memeplex, the worst and _best_ fitness of the frog are denoted as *X*_*w*_ and *X*_*b*_, respectively.
  Step 10: Select the current best fitness as *X*_*g*_.
  Step 11: Improve the frog with the worst fitness according to (4) and (5).

## 5. Application of PSO-SFLA Algorithm in Multipath Transmission of MWSNs

In this paper, we mainly study the routing strategy of MWSNs multipath data transmission and use the shuffled frog leaping algorithm optimized by PSO algorithm to study the optimal alternative routing construction strategy. Sending data from the source node of the sensor network to the destination node is analogous to the process of finding food for the frog group, and the sensor node is analogous to the individual frog population. The optimal path for data transmission is the path for the individual frog to find the best food location. The shuffled frog leaping optimized by PSO algorithm is used to solve the multipath data transmission problem of WSNs, and an efficient and reliable routing transmission path is quickly restored by making full use of the information provided by the original path. The proposed PSO-SFLA algorithm provides faster global convergence performance and more accurate solutions for network optimization. The steps of multipath data transmission based on particle swarm optimization hybrid frog leaping algorithm are as follows.

**Figure 3:**
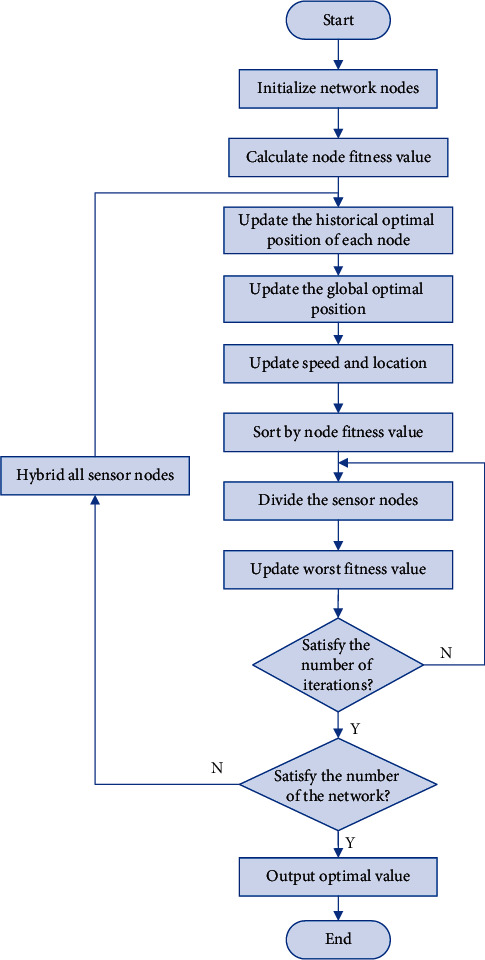
Flow chart of multipath transmission strategy for MWSNs based on PSO-SFLA.

  Step 1: Initialize the sensor network node parameters, including the number of sensor nodes, the dimension of a single node, the number of nodes of a single random network group, the number of sub sensor network groups, the number of iterations of a single random network, the number of mixed iterations of the whole network, and limiting the longest and shortest values of a single path.
  Step 2: Take the sensor continuous variable function of each node as the input, and obtain the fitness value of the sensor node.
  Step 3: Obtain the current individual extreme value of the entire population and the optimal value of the entire sensor network. Compare the fitness value of the sensor node with the current individual extreme value. If the current fitness value is better than the current individual extreme value, the current individual extreme value is set equal to the current fitness value, and the current individual extreme value position is equal to the current position in the dimension space.
  Step 4: Compare the fitness value of the sensor node with the optimal value of the entire sensor network. If the fitness value of the current sensor node is better than the optimal value of the entire sensor network, it is reset to the current value.
  Step 5: Change the velocity and position of the sensing node according to (4) and (5), respectively.
  Step 6: When the number of iterations of the whole network reaches the maximum number or meets the minimum error requirement, stop the iteration; otherwise, jump to Step 2.
  Step 7: Search for possible solutions to the population of the frogs, filter the optimal node and the worst node and rank the moderate value of each sensor node in descending order, and store it. Divide each sensor node into each subsensor network in a regular order to form *N* subsensor networks. The probability that each sensor node is selected to enter the subsensor network.
  Step 8: The sensor network node *i* is denoted as *X*_*i*_=(*X*_*i*1_, *X*_*i*2_,…*X*_*is*_), where the parameter *S* represents the number of variables.
  Step 9: For each subsensor network, determine the optimal value, the worst value of the subsensor network node and the optimal value of the entire sensor network. The fitness value of a single sensor node is updated according to (8), and it is repeated for each subsensor network.
  Step 10: Improve the sensor node with the worst fitness according to (4) and (5). When the subsensor network completes its iteration times and conforms to the full network iteration times, the update ends and the optimal value of the entire network is output. Otherwise, mix all sensor nodes and return to the sorting step for reranking calculation.  Figure 3 shows the workflow of the multipath transmission of MWSNs based on PSO-SFLA proposed in this paper.

In this paper, a multipath data transmission model for MWSNs is established, and an optimal alternative route recovery strategy based on particle swarm optimization hybrid frog leaping algorithm is proposed. The proposed algorithm further improves the exploration and development ability of the algorithm, avoids premature convergence caused by the algorithm falling into local optimum, and improves the optimization efficiency and performance. The proposed algorithm provides a stable and reliable data transmission environment in the multipath transmission strategy of MWSNs, an efficient multipath route recovery mechanism, improves the robustness and reliability of the network, and prolongs the lifetime of the network.

## 6. Algorithm Comparison and Result Analysis

To test the performance of MWSNs multipath transmission of the algorithm proposed in this paper, the performance simulation experiment was carried out with MATLAB 2014b software. The sensor nodes are randomly and uniformly distributed in a two-dimensional space of 500 × 500 m^2^. During the network rounds simulation process, the source node generates 10 data packets to send data to the destination node of Sink, and the size of each data packet is 4 kb. The total energy consumption of the sink is set to 500 J, regardless of its energy consumption, and it moves in a straight line at a constant speed of 1 m/s. The parameters of the hybrid frog leaping algorithm are set as follows: the total number of frog population *P* is 100, the number of subpopulations is 20, and the number of frog individuals in each subpopulation is 10. The maximum number of evolutionary iterations within the subpopulation is 10, the global maximum number of evolutionary iterations is 50, the dimension of the optimal solution space is 20, and the algorithm runs 30 times independently with a population of 50. The parameters of particle swarm optimization are set as follows: the population number is 20, the inertia weight *w* = 0.96, *c*_1_ = 0.5, and *c*_2_ = 0.7, and the number of iterations is 50. The simulation environment parameter settings are shown in Table 1.

We compare the proposed algorithm with PSO and basic SFLA algorithm and make detailed comparisons in terms of multipath transmission effect comparison, network energy consumption, packet loss rate, transmission delay, network connectivity, and network reliability.

### 6.1. Comparison of Multipath Transmission Renderings

**Figure 4:**
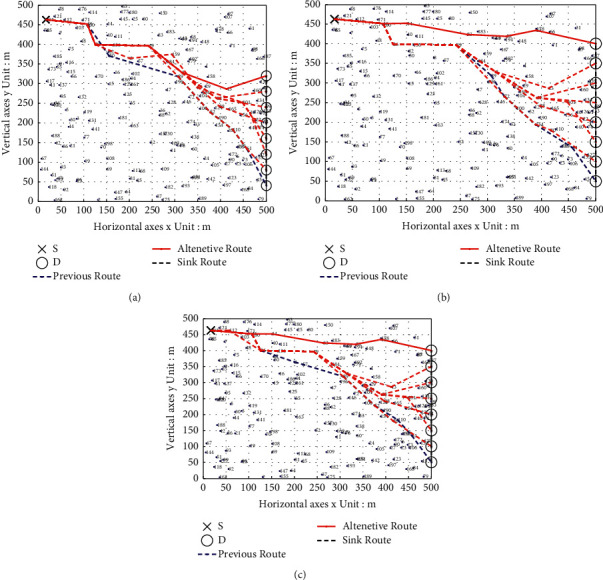
Moving path transfer path of mobile sink (8 times). (a) PSO. (b) SFLA. (c) PSO-SFLA.

**Figure 5:**
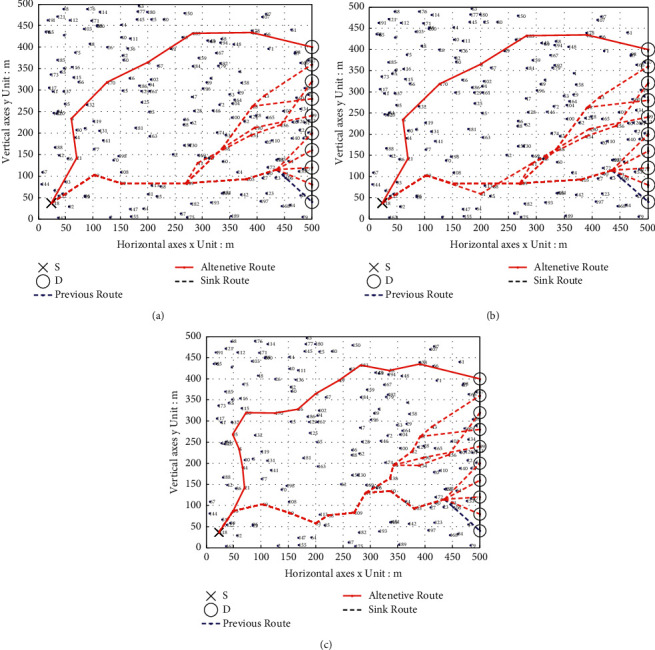
Moving path transfer path of mobile sink (10 times). (a) PSO. (b) SFLA. (c) PSO-SFLA.

To better reflect the effectiveness of the route recovery strategy algorithm adopted in this paper, in the simulation process, we give the uniform linear motion of the sink node and the constantly changing transmission path of the source node. Figures 4 and 5 show the comparison of path restoration effect graphs for the three algorithms with 100 nodes and 200 nodes.

As can be seen from Figure 4, the source node we set is the upper left corner of the graph, numbered 191. The sink moves in a straight line at a constant speed, moves eight times to collect data, and waits for the data sent by the source node 191. As the sink moves in a straight line at a constant speed, it can be seen that the source nodes of the three multipath data transmission algorithms will quickly establish an alternative path to send the sensed data to the mobile sink, but the lengths of the constructed transmission paths are different. When the mobile sink node starts to move, the multipath transmission strategy of the PSO protocol in the source node 191 to the destination node sink is 191 ⟶ 100 ⟶ 50 ⟶ 89 ⟶ 52 ⟶ 110 ⟶ 61 ⟶ sink, with a total of seven hops. The SFLA protocol multipath transmission strategy is 191 ⟶ 100 ⟶ 26 ⟶ 49 ⟶ 89 ⟶ 72 ⟶ 154 ⟶ 61 ⟶ sink, with a total of eight hops. The multipath transmission strategy of the PSO-SFLA protocol is 191 ⟶ 112 ⟶ 26 ⟶ 89 ⟶ 52 ⟶ 110 ⟶ 61 ⟶ sink, with a total of seven hops. When the mobile sink node moves to the eighth time, the multipath transmission strategy of the PSO protocol is 191 ⟶ 46 ⟶ 26 ⟶ 49 ⟶ 1 ⟶ 43 ⟶ sink, a total of six hops. The SFLA protocol multipath transmission strategy is 191 ⟶ 46 ⟶ 14 ⟶ 83 ⟶ 56 ⟶ 178 ⟶ sink, with a total of six hops. The multipath transmission strategy of the PSO-SFLA protocol is 191 ⟶ 46 ⟶ 14 ⟶ 83 ⟶ 56 ⟶ 178 ⟶ sink, with a total of six hops. The multipath transmission strategy of the PSO-SFLA protocol is 191 ⟶ 46 ⟶ 14 ⟶ 83 ⟶ 56 ⟶ 178 ⟶ sink, with a total of six hops.

The source node set in Figure 5 is the lower left corner of the graph, numbered as 118 nodes. When the mobile sink node starts to move, the multipath transmission strategy of the PSO protocol in the source node 118 to the destination node sink is 118 ⟶ 19 ⟶ 60 ⟶ 109 ⟶ 105 ⟶ 172 ⟶ sink, with a total of six hops. The multipath transmission strategy of SFLA protocol is 118 ⟶ 19 ⟶ 69 ⟶ 109 ⟶ 105 ⟶ 172 ⟶ 61 ⟶ sink, with a total of seven hops. The multipath transmission strategy of the PSO-SFLA protocol is 118 ⟶ 93 ⟶ 19 ⟶ 69 ⟶ 81 ⟶ 68 ⟶ 109 ⟶ 11 ⟶ 60 ⟶ 24 ⟶ 105 ⟶ 172 ⟶ sink, with a total of 12 hops. When the mobile sink node moves to the 10th time, the multipath transmission strategy of the PSO protocol is 118 ⟶ 21 ⟶ 5 ⟶ 170 ⟶ 8 ⟶ 183 ⟶ 12 ⟶ sink, with a total of seven hops. The SFLA protocol multipath transmission strategy is 118 ⟶ 21 ⟶ 5 ⟶ 170 ⟶ 8 ⟶ 183 ⟶ 12 ⟶ sink, with a total of seven hops. PSO-SFLA protocol multipath transmission strategy is 118 ⟶ 93 ⟶ 21 ⟶ 44 ⟶ 5 ⟶ 95 ⟶ 56 ⟶ 170 ⟶ 16 ⟶ 8 ⟶ 49 ⟶ 183 ⟶ 58 ⟶ 12 ⟶ sink, with 14 hops in total.

Compared with the other two algorithms, it can be found that the data transmission alternative path of the PSO-SFLA algorithm proposed in this paper has long hops, but there are no more twists and turns. Try to find the shortest path with the shortest data transmission delay, which balances the network energy consumption; the network performance is the best and the reliability is the highest. The main reason is that after the route recovery strategy algorithm in this paper replaces the path, it will replan the shortest path, and there are few paths that overlap with the previous data transmission. In addition, the algorithm proposed in this paper is not only limited to the original shortest path but researches the shortest path according to the surviving nodes to find the global best path.

### 6.2. Comparison of Network Energy Consumption

**Figure 6:**
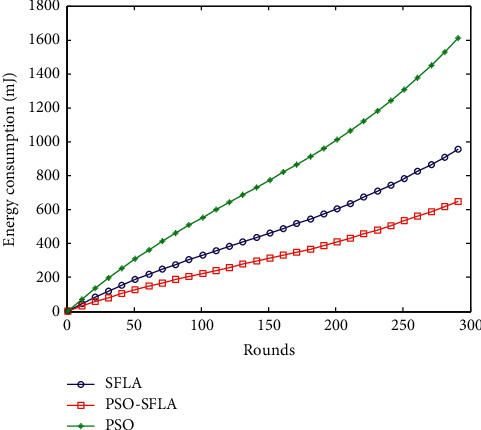
Comparison of network energy consumption.

Network energy consumption is an important parameter to evaluate the network lifetime of MWSNs, which directly affects the network lifetime.  Figure 6 shows the comparison of network energy consumption of the three algorithms. The horizontal axis in Figure 6 is the number of simulated polls, and the vertical axis is the overall energy consumption of the network.

From the comparison of the three algorithms in Figure 6, it can be clearly seen that with the increase of the number of simulation rounds, the network energy consumption of the three algorithms is gradually increasing, and there is a certain linearity. Among them, the network energy consumption of the PSO algorithm increases linearly, and the slope is the largest. The energy consumption of the basic SFLA algorithm is significantly lower than that of the PSO algorithm. The PSO-SFLA algorithm proposed in this paper has the lowest energy consumption and the smallest growth slope. It can be seen that the optimization of network energy consumption in the multipath transmission process proposed in this paper is very effective.

### 6.3. Comparison of Energy Utilization

**Figure 7:**
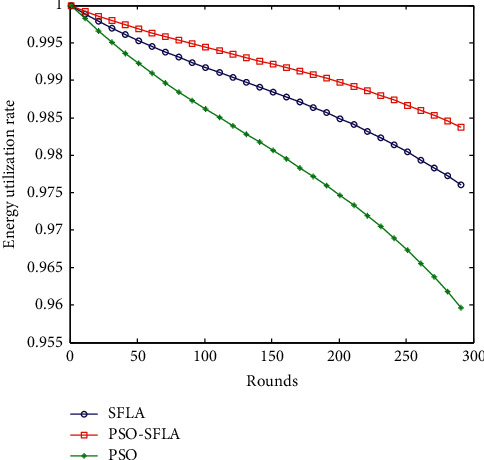
Comparison of energy utilization.

To reflect the importance of the parameter index of network energy, the three multipath data transmission protocols in Figure 7 are in the case of 200 sensor nodes. Comparison of network energy utilization in each round of polling (network utilization is the ratio of the average remaining energy of all nodes to the initial total energy of the network). The horizontal axis of Figure 7 represents the current polling times, and the vertical axis is the energy utilization rate of the sensor network.

The larger the energy utilization value, the better the network energy utilization. From Figure 7, you can see that the network energy utilization rate of the PSO algorithm gradually decreases linearly, and the magnitude is very large. When the node simulation reaches 200 polls, many deaths occur. The energy utilization rate of the basic SFLA algorithm is better and the decrease is smaller. The PSO-SFLA algorithm proposed in this paper has the best energy utilization and the smallest decrease.

### 6.4. Comparison of the Packet Loss Rate

**Figure 8:**
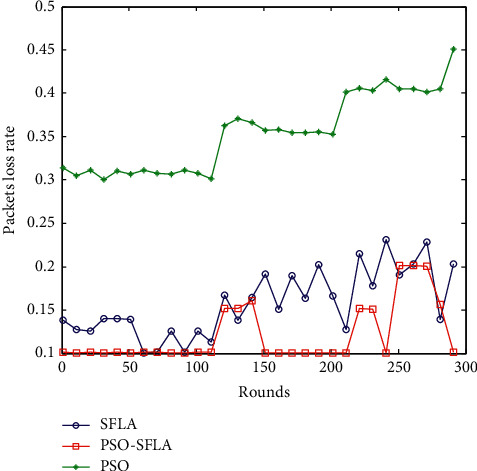
Comparison of packet loss rates.

The packet loss rate is one of the important indicators to evaluate the network performance, which reflects the reliability performance of the network. The packet loss rate refers to the ratio of the number of lost packets to the number of packets normally received by the destination node during the process of sending packets from the sensor node to the destination node. In the case of different numbers of sensor nodes, the comparison of the packet loss rate between the three multipath transmission strategies of the PSO algorithm, the basic SFLA algorithm, and the PSO-SFLA algorithm proposed in this paper is given, as shown in Figure 8.

It can be clearly seen from Figure 8 that the PSO algorithm is very different from the basic SFLA algorithm and the PSO-SFLA algorithm proposed in this paper. Its packet loss rate is always between 0.3 and 0.45, and the overall network packet loss rate is very large. The packet loss rate of the basic SFLA algorithm is between 0.1 and 0.25, and the fluctuation range is large. The packet loss rate of the PSO-SFLA network proposed in this paper is about 0.1, and the fluctuation range is not large. The proposed PSO-SFLA algorithm has very little packet loss during data transmission, and the network has better reliability and stable performance.

### 6.5. Comparison of Data Transmission Delay

The end-to-end data transmission delay refers to the average time it takes for a data packet to travel from the source node to the destination node. The data transmission delay mainly includes the route discovery delay, the waiting delay at the connection, the transmission delay, and the data retransmission delay of the MAC layer. The real-time performance of the protocol is measured by the transmission delay of successfully received packets. A data is denoted as *T*_*s*_ at the time of transmission of the source node and denoted as *T*_*r*_ at the time of reception of the sink node. Then, the average transmission delay formula is

(10) $$\begin{array}{c} T_{\mathrm{trans}}=\frac{1}{N_{r}}\sum_{i=1}^{v}\left(T_{ri}-T_{si}\right). \end{array}$$

**Figure 9:**
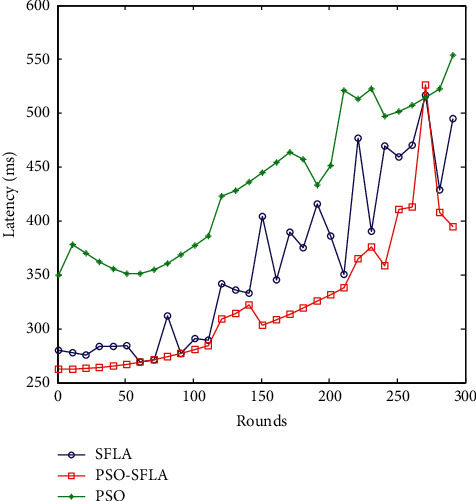
Comparison of network transmission delay.

Among them, the parameter *N*_*r*_ is the total number of successfully received packets.  Figure 9 shows the average packet transmission delay from the source node to the mobile sink simulation for 50 times.

From the comparison of transmission delays in Figure 9, the transmission delays of the three algorithms all increase with time. This is mainly because, with the progress of the simulation, after the energy consumption of the node is exhausted, the number of transmission hops of the node increases, and the transmission delay increases. From the trend of the three algorithms, with the increase of the number of simulation rounds, the transmission delay of the PSO algorithm is the longest compared with the two other algorithms. The basic SFLA algorithm and the proposed PSO-SFLA algorithm are not much different.

### 6.6. Comparison of Network Connectivity

For the dynamically changing mobile sensor network, the commonly used method adopts the method of continuous motion discretization to calculate the connectivity of the network; that is, in a relatively short period of time, it is considered that the network topology does not change, and the network structure remains unchanged. For the network at a certain moment, the calculation of the connectivity rate of MWSNs is generally determined by the perceptual node traversal method. Assuming that a sensing node is used as a reference, the nodes connected from its first hop, second hop, and third hop are sequentially searched until the number of nodes connected to the initial sensing node no longer increases. The mathematical calculation formula of the connectivity ratio *N*_con_ is

(11) $$\begin{array}{c} N_{\mathrm{con}}=\frac{N_{l}}{n}. \end{array}$$

**Figure 10:**
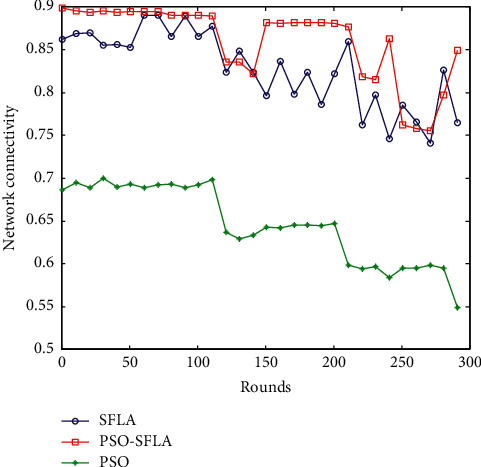
Comparison of network connectivity.

Among them, the parameter *N*_*l*_ is the number of adjacent nodes within the communication range of the node, and the parameter *n* is the number of all nodes in the entire sensor network. The network connectivity comparison of the three algorithms is shown in Figure 10.

tIt can be seen from Figure 10 that, with the increase of the number of simulation rounds, the network connectivity rate of the duo path out of the transmission strategy of the PSO algorithm is low and fluctuates greatly, ranging from 0.55 to 0.72. The network connectivity of the basic SFLA algorithm is relatively high and stable, ranging from 0.75 to 0.9. The PSO-SFLA algorithm proposed in this paper has the highest network connectivity and is stable, but the fluctuation range is a bit large in some places, ranging from 0.8 to 0.9. Overall, the PSO-SFLA algorithm proposed in this paper has the best network connectivity.

### 6.7. Comparison of Comprehensive Reliability of Network

The comprehensive reliability of the network generally calculates the reliability matrix according to the distance between the sensing nodes of MWSNs and then refers to the obtained reliability matrix and random edge reliability matrix samples [36]. According to Monte Carlo analysis, the average node connectivity reliability value after 50 rounds is obtained [37]. The comprehensive network reliability *R*_net_ is composed of network node connectivity reliability *I*_1_, network connectivity rate *I*_2_, and network capacity *I*_3_. Its mathematical formula is

(12) $$\begin{array}{c} \text{Rnet}=\text{0.}3I1+\text{0.}4I2+\text{0.}3I3\text{.} \end{array}$$

**Figure 11:**
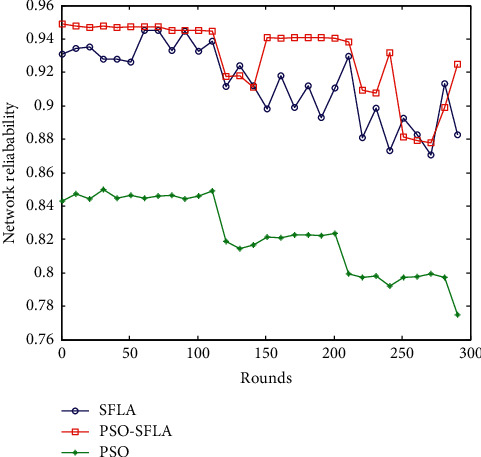
Comparison of network reliability.

The parameter *I*_1_ refers to the reliability of the interconnection between the end-to-end nodes, and the parameter *I*_2_ is the network connectivity rate calculated previously. The network capacity *I*_3_ is the network survival probability. Generally, the ratio of the current network node survival nodes to the number of all network nodes is the network survival probability. The network reliability comparison of the three algorithms is shown in Figure 11.

It can be seen that, with the increase of the number of simulation rounds, the comprehensive network reliability of the multipath transmission strategy of the PSO algorithm gradually decreases, and the fluctuation is large, and the comprehensive network reliability ranges from 0.75 to 0.85. The network comprehensive reliability of the basic SFLA algorithm is relatively stable, with a small fluctuation range, ranging from 0.84 to 0.9. The comprehensive reliability of the proposed PSO-SFLA algorithm has a small fluctuation range, ranging from 0.9 to 0.95, which is slightly higher than the performance of the basic SFLA algorithm. It can be seen that the reliability of the proposed PSO-SFLA algorithm is the highest, which is consistent with the simulation expectations. In the comparison of these three algorithms, the proposed PSO-SFLA algorithm has the highest energy utilization efficiency, the lowest energy consumption, the smallest delay, and the best reliability in the multipath data transmission process of mobile wireless sensor networks.

### 6.8. Comparison of Network Load Balancing

**Figure 12:**
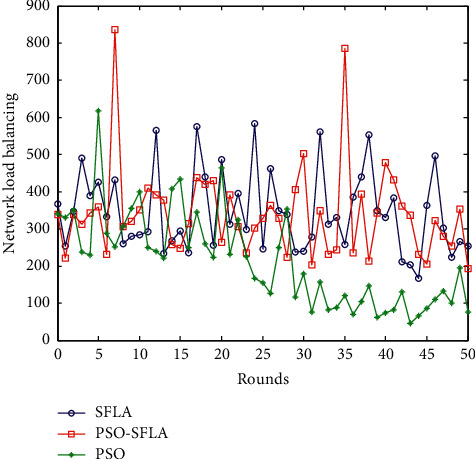
Comparison of network load balancing.

The calculation formula of network load balancing is in [38]. According to the calculation in [38], we can obtain the network load balancing performance of the four algorithms. The comparison of network load balancing is shown in Figure 12.

The higher the number of network balance, the better the network balance. From the comparison of network balance in Figure 12, the network balance of the SFLA algorithm and the shuffled frog leaping algorithm optimized by PSO algorithm is better, and the network balance of the PSO algorithm is poor. This is mainly because the particle swarm algorithm is very easy to fall into a local optimum in the process of finding the optimal solution, and the data transmission path it is looking for is not the optimal solution, resulting in an unbalanced network. The PSO-SFLA algorithm proposed in this paper has a wide search range, is not easy to fall into local optimum, and has the best performance.

## 7. Conclusions

In this paper, the improved SFLA algorithm is combined with the multipath transmission mechanism of mobile wireless sensor networks, which has been improved to a certain extent in terms of the problems affecting the quality of data transmission such as node failure and link failure, which not only ensures the effective transmission of data, but also reduces the energy consumption of nodes accordingly. In this paper, the SFLA algorithm optimized by PSO is proposed to optimize the multipath data transmission of each node, and the node mutation factor is combined into it, which is more in line with the actual changeable complex environment, and can find the shortest and suitable path for data transmission in the process continuous movement of sink. According to the simulation experiments, the particle swarm optimization leapfrog algorithm is significantly better than the particle swarm optimization algorithm and the basic hybrid frog leaping algorithm in terms of network energy consumption, transmission delay, connectivity rate, reliability, and other indicators.

In the future work, the latest swarm intelligence optimization, such as the butterfly algorithm, the dragonfly algorithm, and the sparrow search algorithm, will be adopted and applied to the multipath data transmission mechanism of the heterogeneous mobile wireless sensor network. The next research direction is to quickly search for a transmission path with low latency, low power consumption, and high connectivity and reliability in complex industrial application environments.

## Figures and Tables

**Table 1:** Simulation environment parameter settings.

Variable	Numerical value
Network range	500 × 500 m^2^
Number of nodes	200
Communication radius	80 m
*V*_Sink_	1 m/s
Initial energy	1 J
*E*_elec_	50 nJ/bit
*E*_*fs*_	10 pJ/bit/m^2^
*E*_*mp*_	0.0013 pJ/bit/m^4^
*L*	4000 bits
*d*_0_	$\sqrt{E_{fs}/E_{mp}}=87\text{ m}$

## Data Availability

The data used to support the findings of this study are available from the corresponding author upon request.
